# Retinal nerve fibre layer assessment in myopic glaucomatous eyes: comparison of GDx variable corneal compensation with GDx enhanced corneal compensation

**DOI:** 10.1136/bjo.2007.134080

**Published:** 2008-09-18

**Authors:** S Morishita, T Tanabe, S Yu, M Hangai, T Ojima, H Aikawa, N Yoshimura

**Affiliations:** Department of Ophthalmology and Visual Sciences, Kyoto University Graduate School of Medicine, Kyoto, Japan

## Abstract

**Aim:**

To compare the results of scanning laser polarimetry (GDx) with variable corneal compensation (VCC) and enhanced corneal compensation (ECC) when applied to myopic glaucomatous eyes.

**Methods:**

Forty glaucoma eyes with moderate myopia (between −3 and −6 D) and 35 glaucoma eyes with high myopia (−8 D or greater) were enrolled in this study. GDx VCC, GDx ECC and standard automated perimetry (SAP) were performed. The prevalence of an atypical retardation pattern (ARP), the typical scan score (TSS) and retinal nerve fibre layer (RNFL) thickness were compared between VCC and ECC in both groups of myopic subjects. A correlation analysis between RNFL thickness and visual sensitivity was also conducted.

**Results:**

In both myopic groups, the mean TSS is significantly lower (p<0.0001), and the prevalence of ARP was significantly higher (p<0.0001) by VCC scans than by ECC scans. Temporal, superior, nasal, inferior, temporal (TSNIT) average and temporal average thickness showed significantly higher values (p<0.001) by VCC than by ECC. A statistically significant association was observed between TSNIT average and mean deviation of SAP by ECC scan.

**Conclusions:**

ECC scans showed a better retardation pattern and structure–function relationship than did VCC, and ECC appeared to be more suitable for RNFL assessment in glaucomatous eyes that are moderately to highly myopic.

Myopia is one of the risk factors for glaucoma,^1^ ^2^ so the diagnosis and observation of glaucoma in myopic eyes among the Japanese are extremely important because of the high prevalence of myopia in Japan.^3^ In general, a glaucomatous change is determined by assessment of glaucomatous excavation of the optic disc and by a visual-field defect. However, evaluating the glaucomatous change in myopic eyes is sometimes difficult because the change in the optic disc and in the visual field can be modified by the myopic change itself, especially in eyes that are highly myopic.^4^

A defect in the retinal nerve fibre layer (RNFL) is another characteristic of glaucomatous eyes. Until recently, evaluation of these defects was discussed only qualitatively, but the development of new imaging devices has made it possible now to assess defects in the RNFL quantitatively.^5^^–^7 Among the devices that are currently available for RNFL assessment, scanning laser polarimetry (SLP), that is GDx (GDx Nerve Fiber Analyzer; Carl Zeiss Meditec, Dublin, CA), estimates the thickness of the peripapillary RNFL by measuring the amount of retardation of a polarised laser beam that is caused by the birefringent properties of microtubules within the RNFL.^8^ ^9^ Although retardation is considered to be proportional to the thickness of the RNFL,^10^ it can be affected by artefacts from other ocular tissues; this interferes with accurate assessment of RNFL thickness. Accordingly, GDx with variable corneal compensation (GDx VCC) was developed for better compensation of corneal birefringence and had better diagnostic ability than did GDx with fixed corneal compensation (FCC).^7^ ^11^ Nevertheless, there is still some interference from the subretinal structures,^12^ and the image produced by GDx VCC sometimes shows an atypical retardation pattern (ARP). ARP is seen as alternating peripapillary circumferential bands of low and high retardation, areas of high retardation arranged in a spokelike pattern, or high retardation on the nasal and temporal side of the disc.^13^ ^14^ ARP is thought to be common in eyes with a low signal-to-noise ratio resulting from thinning of the retinal pigment epithelium, such as in aged or myopic eyes.^13^ ^15^

Enhanced corneal compensation (ECC), which is a newer scheme based on the compensation method, has been developed to improve the signal-to-noise ratio and to eliminate the artefacts that are associated with ARP.^16^ The ECC algorithm introduces a predetermined large birefringence bias in order to shift measurement of total retardation to a higher value region, thus removing noise and circumventing the problem of ARP. Several studies have demonstrated the benefits of GDx ECC for the assessment of the RNFL in eyes with ARP.^16^^–^19 However, to our knowledge, there is no report describing the use of GDx ECC for measurement of the RNFL in glaucomatous eyes that are myopic, and little is known about the feasibility of GDx in such eyes.

To determine whether or not RNFL analysis by GDx might be helpful for the quantification of glaucomatous changes in myopia, we compared the use of VCC with that of ECC in glaucomatous eyes with moderate and with high myopia.

## METHODS

This retrospective cross-sectional study included eyes with open-angle glaucoma of patients who were under age 60, met the inclusion criteria and underwent all tests between March 2006 and December 2006. All patients were evaluated at the Kyoto University Hospital (Kyoto, Japan) and were selected retrospectively from the research database. The Ethics Committee of Kyoto University Graduate School of Medicine approved all protocols, and the procedures adhered to the tenets of the Declaration of Helsinki.

Each subject underwent a comprehensive ophthalmic examination, including best corrected visual acuity testing, slit-lamp biomicroscopy, intraocular pressure (IOP) measurement with Goldmann applanation tonometry, gonioscopy and dilated funduscopic examination using a superfield fundus lens; a review of the medical history was also conducted. To be included, subjects had to be phakic and to have a best corrected visual acuity of 20/40 or better and a myopic spherical equivalent refractive error that exceeded −3.0 dioptres (D). To exclude the myopia due to cataract, eyes with coexisting cataract were not included in the current study. The subjects had to have primary open angle glaucoma or normal tension glaucoma, which was defined as having glaucomatous optic nerve change with corresponding glaucomatous visual-field defect and normal open angle in the absence of other known causes of the disease. The eyes with, retinal disease, uveitis or non-glaucomatous optic neuropathy were excluded from the investigation. To ensure that a myopic chorioretinal change did not affect the visual-field results, eyes with myopic chorioretinal atrophy or a staphyloma were also excluded.

The visual field was analysed by a Humphrey Field Analyzer II (HFA) (model 750, Zeiss-Humphrey Systems, Dublin, CA) using the 30–2 Swedish Interactive Threshold Algorithm (SITA) standard test program. The HFA data had to be reliable, with a fixation loss <25% and false positives and false negatives not exceeding 25%. Glaucomatous field change was defined as: (1) three contiguous points with p<0.05 (at the 5% level on the pattern deviation plot), with at least one point with p<0.01; (2) corrected pattern standard deviation (CPSD) significant at p<0.05; or (3) outside normal limits on the glaucoma hemifield test, determined at least three times by the 30–2 SITA standard test program. To avoid enrolment of subjects with a myopic visual field change, the patients with enlargement of the blind spot and those with an atypical glaucomatous visual field were excluded. Accordingly, only those subjects with a typical glaucomatous visual-field defect, such as nasal step, Bjerrum scotoma or paracentral scotoma, were enrolled. In addition to the glaucomatous visual-field defect, included subjects had to have glaucomatous optic neuropathy, which was defined as a cupping–disc ratio greater than 0.8, rim thinning, notching or a defect of the RNFL. Based on refraction, the subjects were classified into two groups: moderately and highly myopic groups. Moderate myopia was defined as a spherical equivalent between −3.0 D and −6.0 D, whereas high myopia was defined as spherical equivalent of −8.0 D or greater. The mean elapsed time between the HFA test and SLP was 135 days. The HFA mean deviation (MD) and mean total deviation in the superior or inferior hemifield were assessed in relation to RNFL parameters.

### Scanning laser polarimetry

All patients were examined by scanning laser polarimetry both GDx VCC and GDx ECC. The general principles of GDx have already been described.^11^ In our study, each subject, with pupils undilated, had three consecutive scans by VCC and ECC on the same day, each performed by an experienced operator; the best images were selected for analysis. All selected images were of high quality with a centred optic disc, were well focused and illuminated throughout the image, and were without any motion artefacts. Each image had a quality scan score of 8 or greater. A fixed concentric measurement band with a 2.4 mm inner and a 3.2 mm outer diameter was centred on the optic disc, after which the measurements of peripapillary retardation were conducted. Subjects with peripapillary atrophy (PPA) that extended into the measurement ring were excluded. Retardation was converted to an estimate of RNFL depth by the software that provides 256 RNFL depth estimates within an eight-pixel-wide ring with inner and outer radii of 27 and 35 pixels, respectively.

The GDx VCC and ECC parameters investigated in this study were the typical scan score (TSS) and RNFL thickness values including TSNIT, and the superior, inferior, temporal and nasal average thicknesses. TSS, ranging from 0 to 100, is the value based on a support vector machine analysis that is the parameter for ARP; the lower the score, the more the scan is affected by ARP.^13^ ^16^ Eyes with a TSS value of 60–79 were defined as having mild ARP, while eyes with TSS values below 60 were defined as having severe ARP.^16^ ^17^ The TSNIT average indicates the average of all thickness values within 360° of the calculation circle. The superior and inferior average is the average of thickness values in the superior or inferior 120° of the calculation circle; the temporal average is the RNFL thickness in the temporal 50°, and the nasal average is that of the nasal 70° peripapillary area. For a correlation analysis between RNFL thickness and visual field sensitivity, the average thickness values in the superior or inferior 180° were calculated from the raw RNFL data.

### Statistical analysis

Statistical software (Statview 5.0, SAS Institute, Cary, NC) was used for statistical analyses. The demographic data between moderately and highly myopic eyes were compared using the Mann–Whitney U test and Fisher exact test. The parameters of GDx VCC and ECC within moderately or highly myopic eyes were compared using the Wilcoxon signed rank test or the paired Student t test. Comparisons of the parameters by corresponding GDx device between the two myopic groups were conducted by Mann–Whitney U test or the Student t test. For multiple comparison, a Bonferroni adjustment was conducted. The adjusted type I error was set to 5% divided by the number of tests within each analysis. The difference in prevalence of ARP between each scan method or each myopic group was analysed by the McNemar test or the Fisher exact test, respectively. The relationships between TSS and refractive error, and between VCC- or ECC-related parameters and visual-field parameters, were assessed by the Spearman correlation coefficient. Data are given as mean (SD).

## RESULTS

One eye each of the 75 open angle glaucoma subjects was assigned to the moderately myopic (40 eyes) or highly myopic (35 eyes) group, respectively. The demographic data are summarised in table 1. The mean refractive error for the moderately myopic and highly myopic groups was −4.8 (0.9) D and −9.7 (1.5) D, respectively. Axial length was not analysed because this study is retrospective in nature, and not all of the subjects had the data of axial length. There were no significant differences between the two groups with regard to age, the prevalence of peripapillary atrophy (PPA) with visible sclera, MD or total deviation in the superior or inferior visual fields (table 1). HFA mean deviations of the moderately and highly myopic groups were −8.7 (5.4) dB and −9.7 (5.9) dB, and the percentages of early stage glaucoma subjects (MD ⩾−6 dB) were 32.5 (13/40) and 34.3 (12/35), respectively.

**Table 1:** Clinical characteristics of glaucoma subjects with moderate and high myopia

	Moderate myopia	High myopia	p Value
Subjects (n), range	40	35	
Age (years), range	50.4 (9.0), 37 to 60	47.9 (8.0), 21 to 60	0.082*
Refractive error (dioptres), range	−4.8 (0.9), 23.0 to 26.0	−9.7 (1.5), 28.2 to 214.0	<0.0001*
Prevalence of PPA (%), range	60.0, 44.1 to 73.6	71.4, 54.0 to 83.4	0.339†
Mean deviation (dB), range	−8.7 (5.4), 20.5 to 223.1	−9.7 (5.9), 21.0 to 226.6	0.464*
Superior visual sensitivity (dB), range	−12.9 (9.1), 0.4 to 229.1	−13.0 (10.0), 0.8 to 230.3	0.932*
Inferior visual sensitivity (dB), range	−6.7 (7.7), 1.8 to 228.2	−8.7 (8.8), 0.4 to 232.2	0.303*

The prevalence of PPA is shown with the 95% CI. All values except for the prevalence of PPA were expressed as mean (SD) (range).

*Mann–Whitney U test.

†Fisher exact test.

PPA, peripapillary atrophy.

Tables 2, 3 show a comparison of TSS and the prevalence of ARP between the VCC and ECC scans performed on each myopic group. In both myopic groups, the mean TSS by ECC was significantly higher than that by VCC (Wilcoxon signed rank test, p<0.0001), and the prevalence of mild or severe ARP (TSS value below 80) was significantly lower (McNemar test, p<0.0001) when scanned with ECC (0% in moderately myopic eyes and 8.6% in highly myopic eyes) than when scanned with VCC (35% in moderately myopic eyes and 54.3% in highly myopic eyes). When comparing TSS and the prevalence of ARP between the two myopic groups, highly myopic subjects showed significantly lower TSS on both VCC and ECC (Mann–Whitney U test, p = 0.0064 and p = 0.0019, respectively) than did moderately myopic subjects, and also a higher prevalence of severe ARP on VCC (Fisher exact test, p = 0.003) than did moderately myopic subjects.

**Table 2:** Typical scan score by GDx variable corneal compensation (VCC) and enhanced corneal compensation (ECC) in each myopic group

	VCC	ECC	p Value*
Moderate myopia	85.7 (17.2)	99.5 (1.9)	<0.0001
High myopia	66.2 (31.9)	95.9 (7.4)	<0.0001
p Value†	0.0064	0.0019	

*Wilcoxon signed rank test.

†Mann–Whitney U test.

**Table 3:** Prevalence (%) and 95% CI of Atypical Retardation Pattern (ARP) by GDx variable corneal compensation (VCC) and enhanced corneal compensation (ECC) scans in each myopic group

	Moderate myopia		High myopia	
	VCC % (95% CI)	ECC % (95% CI)	VCC % (95% CI)	ECC % (95% CI)
No ARP	65.0 (48.9 to 77.8)	100 (82.8 to 100)	45.7 (30.3 to 62.2)	91.4 (75.2 to 96.6)
Mild ARP	25.0 (14.5 to 41.2)	0 (0 to 17.2)	14.3 (6.7 to 31.2)	8.6 (3.4 to 24.8)
Severe ARP	10.0 (4.4 to 24.9)	0 (0 to 17.2)	40.0 (25.6 to 57.0)	0 (0 to 19.3)

No ARP = no atypical retardation pattern (TSS value of 80–100); mild ARP = mild atypical retardation pattern (TSS value of 60–79); severe ARP = severe atypical retardation pattern (TSS value below 60).

We then analysed whether the presence of PPA and a visible sclera would affect the relationship between the refractive error and TSS. The correlation analysis in the group subdivided by presence or absence of PPA demonstrated that the group with PPA and a visible sclera showed a significant positive correlation between refractive error and TSS (Spearman correlation coefficient, r = 0.32, p = 0.0277 in VCC; r = 0.41, p = 0.0050 in ECC), while the group without PPA failed to show a significant correlation between refractive error and TSS (Spearman correlation coefficient, p = 0.1596 in VCC; p = 0.8359 in ECC).

A comparison of RNFL thickness between use of VCC and ECC revealed that, in both myopic groups, the TSNIT average and temporal average were significantly higher with VCC than with ECC (p<0.0001 for both averages in the moderately myopic group, and p = 0.0004, p<0.0001, respectively, in the highly myopic group) after Bonferroni correction (α = 0.01, five comparisons), while the superior and inferior average thickness did not differ between VCC and ECC (table 4).

**Table 4:** Comparison of GDx parameters between variable corneal compensation (VCC) and enhanced corneal compensation (ECC)

	Moderate myopia			High myopia		
	VCC	ECC	p Value*	VCC	ECC	p Value*
Total average retinal nerve fibre layer (TSNIT) thickness						
Mean (SD)	42.7 (7.8)	39.5 (6.7)	<0.0001	48.1 (9.6)	44.2 (7.8)	0.0004
Range	28.8 to 58.2	22.6 to 52.2		32.5 to 65.1	27.9 to 57.6	
Superior average						
Mean (SD)	49.6 (12.2)	49.9 (11.6)	0.697	54.5 (12.4)	54.4 (12.8)	0.9165
Range	26.7 to 76.6	23.7 to 77.3		32.5 to 75.9	28.5 to 74.5	
Inferior average						
Mean (SD)	47.1 (9.8)	46.3 (10.1)	0.4607	52.2 (10.2)	52.0 (10.7)	0.8941
Range	26.6 to 65.9	29.8 to 66.5		33.0 to 75.3	29.3 to 72.7	
Temporal average						
Mean (SD)	37.2 (14.9)	20.1 (5.7)	<0.0001	50.9 (27.6)	25.9 (9.6)	<0.0001
Range	13.7 to 67.4	10.8 to 35.8		18.0 to 115.0	11.1 to 49.2	
Nasal average						
Mean (SD)	27.4 (6.9)	24.2 (5.1)	0.0028	28.4 (5.7)	26.8 (7.2)	0.1946
Range	18.5 to 56.7	15.5 to 36.0		21.3 to 50.7	15.9 to 47.7	

*Paired Student t test.

TSNIT, temporal, superior, nasal, inferior, temporal.

To determine if there was a structure–function relationship, the correlation between VCC- or ECC-derived RNFL thickness and the corresponding HFA parameters was assessed by Spearman correlation coefficient (table 5). The TSNIT average showed a significant correlation with corresponding HFA MD, except for the VCC scans of moderately myopic eyes (r = 0.19 for VCC and r = 0.42 for ECC in moderately myopic eyes; r = 0.36 for VCC and r = 0.46 for ECC in highly myopic eyes). When analysing the correlation between hemifield RNFL thickness and total deviation in the corresponding area, superior RNFL thickness and total deviation in the inferior visual field demonstrated a significant correlation (r = 0.38 for VCC and r = 0.50 for ECC in the moderately myopic group; r = 0.54 for VCC and r = 0.60 for ECC in the highly myopic group). In contrast, the correlation between inferior RNFL thickness and total deviation in the superior visual field was significant only by ECC measurement in the moderately myopic group (r = 0.39, p = 0.0112). All correlation values between ECC and visual-field indices were higher than those between VCC and visual field indices.

**Table 5:** Correlation analysis between retinal nerve fibre layer thickness and mean total deviation in corresponding area

	Moderate myopia				High myopia			
	VCC		ECC		VCC		ECC	
	r*	p Value	r*	p Value	r*	p Value	r*	p Value
Total visual field	0.19	0.2470	0.42	0.0068	0.36	0.0312	0.46	0.0048
Superior visual field	0.26	0.1065	0.39	0.0112	0.07	0.6827	0.28	0.1027
Inferior visual field	0.38	0.0155	0.50	0.0008	0.54	0.0007	0.60	<0.0001

*Spearman correlation coefficient.

ECC, enhanced corneal compensation; VCC, variable corneal compensation.

## DISCUSSION

In myopic eyes, it is believed that the assessment of RNFL thickness by SLP should be conducted with caution because of the incidence of ARP. However, there is a paucity of reports that describe the details of SLP assessment of myopic glaucomatous eyes. In the study described herein, we conducted RNFL measurement using both GDx VCC and GDx ECC, and have shown the results of SLP assessment of myopic glaucomatous eyes. As is expected in myopic eyes, we noted that with GDx VCC, myopic glaucomatous eyes, especially highly myopic eyes, showed an ARP in many cases. Although the definition of ARP was not identical between studies, the prevalence of ARP (TSS less than 80) in our study, 14/40 (35.0%) of the moderately and 19/35 (54.3%) of the highly myopic eyes, was higher than that in previous studies, most of which were conducted in populations without highly myopic eyes. According to previous reports, the prevalence of ARP was between 7 and 51%,^13^ ^16^ ^17^ ^19^ and TSS values were between 71.2 and 85.4.^17^^–^19 In myopia, PPA and/or higher degrees of myopia, both of which are possibly related to high backscattering from the sclera, have been suggested to be involved in the high prevalence of ARP. In fact, several studies have reported that RNFL assessment by GDx was strongly affected in the region where PPA with visible sclera was present.^15^ ^20^ ^21^ We also found that in both moderately and highly myopic groups, subjects showing PPA with visible sclera had significantly lower TSS (Mann–Whitney U test, moderately myopic group, p = 0.001; highly myopic group, p = 0.011). On the other hand, few reports discuss the relationship between the degree of myopia and ARP. Only the study by Bagga and colleagues indicated that the presence of ARP was correlated with age, but not with refractive error or with HFA MD.^13^ We found a positive correlation between TSS and refractive error in the subjects with PPA, but in those subjects without PPA, we failed to find a statistically significant correlation between TSS and refractive error. This may indicate that TSS is associated only indirectly, by means of PPA, with refractive error. Although we did not analyse the relationship between refractive error and PPA, primarily because of the difficulty of quantifying the extent of PPA, it is known that the prevalence of large PPA increases in association with higher degrees of myopia.^22^ Further study with a large sample and with quantification of the size and the severity of PPA should clarify the relationship between myopic degree and ARP.

In contrast to VCC, the use of ECC dramatically reduced the presence of ARP. By ECC, all subjects in the moderately myopic group, and 91.4% of highly myopic eyes, showed the typical retardation pattern, while no severe ARP was observed. At the same time, the artefactually high retardation values observed in the temporal peripapillary area by VCC in both myopic groups were eliminated by ECC. The high retardation in the temporal peripapillary area is thought to be a common artefactual RNFL measure by VCC scans. Bagga and colleagues reported that the amount of ARP seen in a study using VCC affected most prominently the temporal peripapillary region, and that TSS was negatively correlated with the temporal RNFL thickness.^13^ Previous studies that compared VCC with ECC indicated that ECC resulted in an improvement in TSS and a reduction in the high retardation values of several peripapillary sectors, including temporal sectors.^18^ ^19^ Furthermore, those studies reported a better relationship between RNFL values and visual-field parameters when using ECC compared with VCC.^23^ ^24^ While our population included only moderately to highly myopic eyes, which is different from the other studies, we also noted the beneficial effects of ECC on ARP and atypical RNFL values and a better structure–function relationship with ECC than with VCC.

Although we found that total average RNFL thickness measured by GDx VCC and ECC in highly myopic glaucomatous eyes was correlated significantly with HFA MD, Melo and colleagues recently reported that neither GDx VCC nor OCT RNFL assessment was useful for discriminating glaucoma in highly myopic eyes.^25^ This difference in results may be due to a difference in study population and to the type of GDx used in the two studies. The spherical equivalent of their population was −14.66 (4.43) D, which is markedly higher than that of our population (spherical equivalent −10.1 (1.7) D). Because the refractive error in their patients was so high, although they did not mention the presence of PPA, most of their subjects were presumed to have a large PPA,^22^ which would have markedly affected the retardation pattern of GDx. Furthermore, they used only VCC, whereas we used ECC as well as VCC. In our highly myopic population, while less than half of the subjects showed a normal retardation pattern by VCC, most subjects (91.4%) showed a normal retardation pattern by ECC. Although further investigation with a larger sample is needed, we believe that GDx is useful for the assessment of the RNFL, even in highly myopic glaucomatous eyes if cases with a good image are carefully selected or they are imaged by ECC.

In conclusion, we found a high prevalence of ARP using GDx VCC assessment, especially for highly myopic glaucomatous eyes. However, the use of GDx ECC significantly improved ARP, even in highly myopic eyes, and led to a better structure–function relationship. These findings suggest that GDx ECC is more suitable for the assessment of RNFL thickness in moderately to highly myopic glaucomatous eyes.
